# Quality of life and functional capacity of elderly women with knee osteoarthritis

**DOI:** 10.1590/S1679-45082013000200013

**Published:** 2013

**Authors:** Janice Chaim Alves, Debora Pastore Bassitt

**Affiliations:** 1 Instituto de Assistência Médica ao Servidor Público Estadual, São Paulo, SP, Brazil

**Keywords:** Quality of life, Osteoarthritis, knee, Aged, Health of elderly, Chronic disease

## Abstract

**Objective:**

To correlate functional ability and quality of life of elderly women with knee osteoarthritis.

**Methods:**

Cross-sectional study composed of 40 elderly women with knee osteoarthritis. We used the following instruments: identification questionnaire, Western Ontario and McMaster Universities Arthritis Index (WOMAC), and World Health Organization Quality of Life Questionnaire-OLD (WHOQOL-OLD). The significance level was 0.05 (5%), and confidence intervals were 95%. For statistical analysis we used parametric statistical tests, descriptive analysis, test for equality of two proportions, Pearson's correlation, correlation test, and analysis of variance.

**Results:**

The mean age (± standard deviation) was 74.1 (±6.7) years, and 47.5% of patients had osteoarthritis in both knees. Moderate pain was reported by 45% of patients when they walked on a flat plane and 40% when they were seated or lying down; 55% had severe or very severe pain when climbing or descending stairs; 50% reported moderate joint stiffness after sitting, lying, or resting; and 65% reported moderate or little stiffness after waking. In physical function, 60% of patients had moderate or severe difficulty in descending stairs and 67.5%, when climbing stairs; 60% reported severe or very severe difficulty in getting in and out of the car, and 70%, when performing strenuous housework. The correlation with WHOQOL-OLD and WOMAC was negative and not significant except for autonomy, which was significant. Sedentary and elderly women who used walking aid devices had worse WOMAC functional capacity, but this finding was not statistically significant. In WHOQOL-OLD, volunteers scored higher on social participation and engagement in physical activity on autonomy, which was statistically significant compared with the nonvoluntary and sedentary domains, respectively.

**Conclusion:**

It is possible to have a good quality of life even with functional impairment from knee osteoarthritis.

## RESUMO

**Objetivo:**

Relacionar capacidade funcional e qualidade de vida de idosas com osteoartrite do joelho.

**Métodos:**

Estudo transversal, envolvendo 40 idosas com osteoartrite do joelho. Foram aplicados os seguintes formulários: Questionário de Identificação, Questionário para Osteoartrite *Western Ontário and McMaster Universities* e Questionário de Qualidade de Vida da Organização Mundial da Saúde (WHOQOL-OLD, sigla em inglês). Definiu-se nível de significância de 0,05 (5%) e intervalo de confiança de 95%. Foram utilizados testes estatísticos paramétricos, análise descritiva, teste de igualdade de duas proporções, correlação de Pearson, teste de correlação e teste de ANOVA.

**Resultados:**

Com idade média de 74,1 (±6,7) anos, 47,5% dos pacientes tinham osteoartrite nos dois joelhos. Apresentaram dor moderada: 45% ao caminhar em lugar plano; 40% sentando-se, deitando-se e deitado; 55% apresentaram dor intensa ou muito intensa ao subir ou descer escadas; 50% relataram rigidez articular moderada após sentar, deitar e repousar; e 65% relataram pouca ou moderada rigidez após acordar. Na função física, 60% apresentaram dificuldade moderada ou intensa para descer escadas; 67,5% para subir escadas; 60% relataram dificuldade intensa ou muito intensa para entrar e sair do carro; e 70% para fazer tarefas domésticas pesadas. A correlação do WHOQOL-OLD com o Questionário para Osteoartrite *Western Ontário and McMaster Universities* foi negativa e não significativa – exceto em “autonomia”, que foi significativa. Idosas sedentárias e que usam dispositivo de auxílio à marcha apresentaram pior capacidade funcional no Questionário para Osteoartrite *Western Ontário and McMaster Universities*, sem significância estatística. No WHOQOL-OLD, voluntárias tiveram maior pontuação em “participação social” e praticantes de atividade física, em “autonomia”, com diferença estatisticamente significante em relação às não voluntárias e às sedentárias, respectivamente.

**Conclusão:**

É possível ter uma boa qualidade de vida mesmo com o comprometimento funcional gerado pela osteoartrite do joelho.

## INTRODUCTION

Osteoarthritis (OA), also called osteoarthrosis or, simply, arthrosis, is the most common musculoskeletal condition. It affects 10% of the global population older than age 60 years^(1)^.

Clinical and functional changes caused by OA can influence the quality of life (QL) of elderly people with this disease^(2)^. Women with OA of the knee often report more pain and functional loss and commitment of QL than men ^(3)^.

QL has been defined several times through the years. World Health Organization (WHO) researchers investigating QL, known as the WHOQOL group, proposed a multidimensional subjective definition that includes positive and negative elements. They define QL as “the perception of the individual of their position in life in the context of the culture and value systems in which they live and in relation to their goals, expectations, standards and concerns”. From this concept, the WHOQOL group elaborated the WHOQOL-100 instrument and a specific version to evaluate the QL of the elderly population, the WHOQOL-OLD^(4,5)^.

Although studies have assessed the QL of individuals with knee OA^(6,7)^. these studies also cover a large age group and thus use generic questionnaires, with nonspecific concepts for a specific age.

## OBJECTIVE

This study correlated functional capability and quality of life of elderly patients with knee osteoarthritis

## METHODS

This study was approved by the Ethical and Research Commitee of the *Instituto de Assistência Médica ao Servidor Público Estadual* (IAMSPE) protocol number 0131/11. It was a cross-sectional study performed in the Physiotherapy Sector of the Outpatient Service of Physical Medicine of IAMSPE in 2012.

The sample was composed of women aged 60 years or older referred for the first time to the physiotherapy department for treatment of unilateral or bilateral knee OA that had been diagnosed by an orthopedist or physiatrist. The patients could ambulate independently, with or without walking aid devices, and had not undergone any previous knee surgery. Other previous treatments were not considered exclusion criteria in the study. All participants were in the active phase of the disease, which had prompted their referral to physiotherapy. Patients filled out questionnaires on the first day of treatment in the outpatient unit. Before treatment began, the patients were evaluated by the investigator in the physiotherapy department in order to verify that they met the inclusion criteria. Those included were invited to respond to questionnaires before they began treatment.

All participants answered the identification questionnaire, including the following data: age; sex; date of birth; race; marital status; number of children; weight and height; type, frequency, duration, and local practice of physical exercise; participation in leisure group, profession or current occupation; participation in volunteer activities and leisure activities; medicines currently being used; use of walking aid devices; and previously diagnosed diseases and impairments.

We used the Western Ontario and McMaster Universities Arthritis Index (WOMAC) translated and validated in Brazilian Portuguese; this instrument is specific for OA of the knee and hip^(8)^. The instrument included 24 questions divided into three subscales – pain (five questions), joint rigidity (two questions), and a Likert-type scale (none, mild, moderate, strong, and very strong) – scored as 0, 1, 2, 3, and 4, respectively. A score of 0 represented the absence of symptoms and 4, the worst symptoms. Each dimension receives a score that is transformed to a scale ranging from 0 (better health status) to 100 (worse heath status).

To evaluate QL, we used the WHOQOL-OLD instrument, which has a specific module for assessing QL in elderly. This questionnaire has 24 items attributed to six domains: sensorial function, which evaluates vision, auditory function, touch, olfaction, and taste and impact of loss of sensorial abilities in QL; autonomy, which concerns the capability of living autonomously and making decisions; past, present, and future activities, which describe satisfaction with life achievements and desires; social participation, which concerns participation in daily activities and the community; death and dying, which is related to worries, disturbances, and fear of death and dying; and intimacy, which evaluates the capability of having personal and private relationships. Each domain had four items scored from 1 to 5; the scores for all domains ranged between 4 and 20. Scores are combined to produce a total score, which could vary between 24 and 120. A high total score indicated better QL. The score could also be provided in percentages, according to the WHOQOL-OLD manual^(4,5)^. All patients completed the questionnaires.

For statistical analysis, we defined the significance level as 0.05 (5%) and used 95% confidence intervals. We used parametric statistical tests. For quantitative variables of demographic data we performed descriptive analysis. The test for equality and two proportions was used for variables concerning whether the patients had their own home, who they lived with, their marital status, and their race. Pearson correlation coefficient measured the degree of relation between WOMAC scores and body mass index (BMI; body weight in kg divided by height in m^2^); WHOQOL-OLD scores; correlation of total of diseases; and commitment with WHOQOL-OLD scores. To validate correlations we used the correlation test.

The analysis of variance was used to compare WOMAC scores with physical exercise and the use of a walking aid device, as well as to compare mean WHOQOL-OLD scores for the following covariates: volunteer work, participation in leisure group, physical exercise, leisure activities, and use of a walking aid device. We used SPSS software, version 17; Minitab 16; and Excel 2010 for analyses.

## RESULTS

All 40 participants were women, with a mean age (± standard deviation) of 74.1 (±6.7) years. They had an average of 8.5 (±4.9) years of formal education, 2.2 (±1.7) children, monthly personal income of R$ 1.613,00 (±R$ 1.014,00), and monthly family income of R$ 2.889,00 (±R$ 2.333,00). Most lived in their own home (87.5%), and 82.1% lived with one or more people; 40% were widowed; 12.5% were single; and 7.5% were separated or divorced. Fifty percent of patients were white and 23.7% were brown. The mean BMI was 28.35kg/m^2^ (±4.24).

Per the interviews, 5% of patients reported having OA only in the right knee, 15% only in the left knee, and 47.5% in both knees; 30% did not know.

Total self-reported involvement had a mean of 3.5 (±1.8), being positioned between more frequent systemic arterial hypertension, osteoporosis, gastrointestinal disease, spine problems, and visual changes. There was no significant statistical correlation between total number of involvements with WHOQOL-OLD scores.

Among medicines used to treat OA and its symptoms were glucosamine, chondroitin, and paracetamol. Of participants, 50% used no medicines for OA, 20% used one medicine, 15% used two medicines, and 12.5% used three medicines.

According to assessments for pain intensity by WOMAC, moderate pain occurred in 45% patients when they walked on a flat plane and 40% at night when they were seated or went to bed; 55% had intense or extremely intense pain when they ascended or descended stairs. Concerning joint rigidity, 50% of patients reported that they had moderate rigidity when they were sitting, lying down, or resting during the day, and 65% reported little or moderate rigidity after waking up. In relation to physical activity (i.e., the individual's ability to move and take care of himself or herself), 60% had moderate or intense difficulty when descending stairs and 67.5%, when ascending stairs; 60% reported intense difficulty when getting into or out of the car and 70%, when performing strenuous domestic activities.

The WOMAC score was positively correlated with BMI, but this finding was not significant. When we assessed WHOQOL-OLD dimensions after excluding the autonomy domain, the WOMAC was negatively correlated (Table 1). However, when correlations were classified, we did not observe significant changes.

**Table 1:** Correlation between scores of the Western Ontario and McMaster Universities Osteoarthritis Index and the World Health Organization Quality of Life Questionnaire for older people

WHOQOL-OLD dimensions	WOMAC	
	Correlation (%)	p value
Sensorial function	-17.6	0.278
Autonomy	0.5	0.978
Past, present and future activities	-28.6	0.074
Social participation	-24.7	0.124
Death and dying	-20.8	0.199
Intimacy	-34.5	0.029*
Total	-37.0	0.019*

Statistical tests: Pearson correlation (relationship between WOMAC and WHOQOL-OLD scores) and correlation test (correlation validations).

* statistically significant.

WOMAC scores were higher among participants who did not perform physical exercise (53.81±19.23 compared with 45.29±18.53 among those who exercised) and among those who used any walking aid device (55.78±18.21 compared with 49.39±19.52 among those who did not use a walking aid device). These differences were not statistically significant.

**Figure 1:**
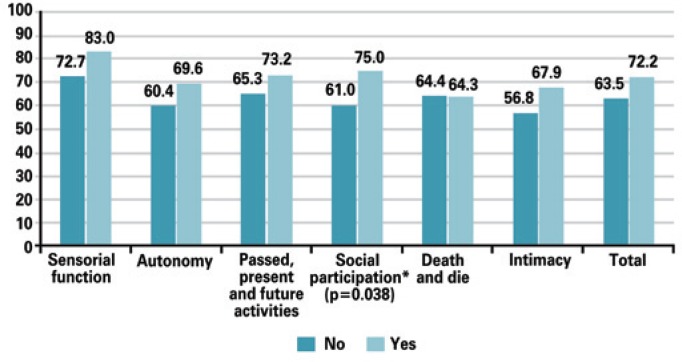
Comparison of quality of life questionnaire score for aging population of the World Health Organization Quality of Life-Old with volunteer work as a variable

**Figure 2:**
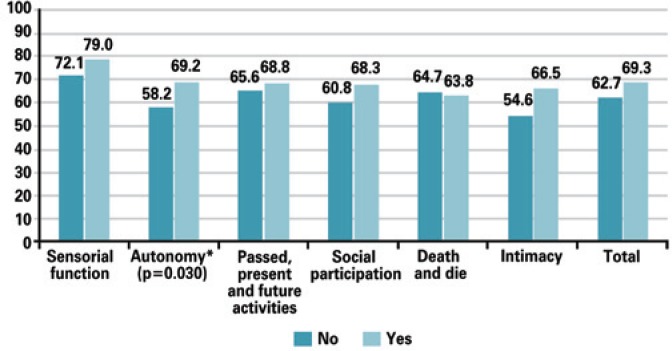
Comparison of quality of life questionnaire score for ageing population of the World Health Organization Quality of Life-Old with “practice of physical exercise” as a variable

In a comparison of mean WHOQOL-OLD scores with the variables volunteer work, participation in a leisure group, physical exercise, leisure activities, and use of a walking aid device, there was a significant statistical difference in the score for social participation; elderly people who volunteered in their community had a mean of 75 (±18.75), compared with 60.98 (±15.07) among those who did not (Figure 1). There was also a significant score for autonomy with physical exercise; patients who exercised had a mean of 69.20 (±12.61), compared with 58.17 (±15.79) for those who were sedentary (Figure 2).

Table 2 shows a descriptive analysis of WOMAC and WHOQOL-OLD scores. The scores varied little, indicating homogenous data.

**Table 2:** Complete descriptive analysis of scores of Western Ontario and McMaster Universities Osteoarthritis Index and the World Health Organization Quality of Life Questionnaire for older people

Descriptive		Mean	Medium	Standard deviation	CV (%)	Q1	Q3	Min.	Max.	n	CI
WOMAC	Pain intensity	53.6	50.0	20.4	38	43.8	65.0	15.0	100	40	0.2
	Rigidity	52.2	50.0	22.4	43	37.5	62.5	12.5	100	40	0.2
	Physical exercise	53.6	54.4	20.3	38	39.3	64.7	14.7	100	40	0.2
WHOQOL-OLD	Sensorial function	74.5	75.0	20.0	27	62.5	87.5	18.8	100	40	6.2
	Autonomy	62.0	62.5	15.5	25	54.7	68.8	25.0	100	40	4.8
	Past, present and future activities	66.7	68.8	15.1	23	56.3	75.0	31.3	100	40	4.7
	Social participation	63.4	68.8	16.4	26	54.7	75.0	25.0	100	40	5.1
	Death and dying	64.4	65.6	24.7	38	48.4	81.3	6.3	100	40	7.7
	Intimacy	58.8	65.6	25.9	44	37.5	75.0	0.0	100	40	8.0
	Total	65.0	65.6	11.7	18	57.0	73.2	39.6	95	40	3.6

Statistical tests: complete descriptive analysis.

CV: coefficient of variation; Q1: 1^st^ quartile; Q3: 3^rd^ quartile; Min.: minimal value; Max.: maximum value; CI: confidence interval.

## DISCUSSION

The age of the studied population differed from that in studies from other countries^(9,10)^ in that our study included only women 60 years of age or older; however, this study population is similar to that in other Brazilians' studies^(2,11)^.

The decision to evaluate only women with knee OA reflects the importance of sex in the study of OA: women have reported more pain and lower QL compared with men, even after adjustment for age differences, severity of OA, and BMI and radiographic differences^(3)^.

When pain, rigidity, and joint and physical function were assessed using the WOMAC, we encountered barriers because of cutoffs to classify the scores obtained from questionnaire. In addition, previous studies were heterogenous with regard to use of WOMAC^(2,12–15)^, which poses difficulties for comparison with our data. Most of our patients reported moderate pain, a finding that agrees with some previous studies^(11,14)^ but not with others^(13,15)^.

More than half of our participants reported intense or extremely intense pain when ascending or descending stairs. Patients also reported moderate joint rigidity. Most patients indicated moderate difficulty with physical function; pain was intense or extremely intense only for ascending and descending stairs, getting in or out of the car, and performing strenuous domestic tasks.

These intermediate points may be attributable to the waiting time between consultation and medical prescription and effective initiation of physiotherapy. Given all the steps involved, from the first consultation in orthopedic unit to undergoing imaging examinations, returning for orthopedic consultation, making an appointment with the physiatrist in the physical medicine unit, scheduling rehabilitation, and attending the first day of physiotherapy, this delay is not surprising. It results from the large demand and the fact that priority for physiotherapy is given to postoperative patients, whose need for return to agility is greater. While waiting to begin treatment, patients sometimes also use analgesics, ear protectors, and home thermotherapy, with or without medical guidance. Thus, many patients attend physiotherapy appointments reporting few symptoms.

The WOMAC was weakly correlated with a BMI that suggested underweight^(16)^, in other words, being underweight was related to worse OA symptoms.

Functional condition was worse among participants who did not practice physical exercise. This raises the question: Is the functional capability of these elderly women affected by a sedentary lifestyle, or do these women avoid exercise because of the severity of OA, with its regular pain and functional damage?

Studies in the literature points out that people with OA often avoid physical exercise because of discomfort, fear of pain, or previous advice to avoid exercise. Patients may also adhere to the false idea that exercise might induce bone and cartilage loss. The fear of movement can affect participation in exercise and social events, thereby increasing physical isolation and social loneliness^(17,18)^.

However, resistance exercises are an important component of general exercise regimen directed to compensate for physical and psychological limitations associated with OA of the knee because it reduces pain, improves physical function, restores muscle and joint mechanical strength, and reduces anxiety and depression. The prescription of exercise is possible across all spectra of OA severity; exercise regimens should take into account pain during and after exercise, incorporate resting periods, and be revised as needed to guarantee adherence to and continuation of exercise^(19)^. The literature contains many studies showing the benefits of physical exercise in knee OA^(20–22)^.

Participants who used walking aid devices had worse functional ability, which corroborates findings of a study showing that the need for such a device is related to aging, greater pain intensity, and incapacity^(23)^. Device use seems to decrease pain, improve function, and improve some aspects of QL in patients with OA^(22,24)^.

In assessment of QL using the WHOQOL-OLD, a high mean score was found for sensorial functioning 75%; this result is similar to that seen in other investigations^(25–27)^. The low mean for intimacy (56.25%) was also similar to other studies^(26,27)^. The lower score for intimacy may be attributable to the fact that 60% of the elderly women were single, widowed, or divorced. Another possibility could be the participants' embarrassment about answering questions concerning their private and intimate relationships.

Studies that used WHOQOL-OLD are still limited, perhaps because it is relatively new. In fact, comparisons with other studies is still extremely difficult.

In correlation analyses between WOMAC and WHOQOL-OLD, worse functional capability was associated with lower QL scores, except in relation to the autonomy domain. It is well known that autonomy is related to the ability to make decisions and act upon them^(28)^. Therefore, physical compromise does not necessarily interfere with patients' decision-making capacity. Even with changes in physical function, autonomy might be perfectly preserved in elderly people.

This study suggests that the elderly women included had QL considered regular to good by the mean score found (65%) and by the fact that WHOQOL-OLD does not have cutoffs to classify; there is only an indication that low scores suggest low QL and that high scores suggest good QL. Investigators on aging affirm that when elderly people define their health as good and reasonable, they do not consider themselves to be without disease; however, they believe that even with their diseases, they can function well in their environment^(28)^.

Elderly women who performed volunteer work had higher means compared with those who did not volunteer, with a statistical difference in the score for social participation. Studies in the literature have also found that volunteer work is related to better QL^(29)^. This corroborates the assertion of the Pan American Health Organization^(30)^ that volunteer work has an important role in maintenance of well-being and QL in the elderly. This organization also posits that volunteering is a key element of active aging in terms of being physically active and involves all types participation: economical, cultural, spiritual, civil, and social.

Limitations of this study include the small number of participants; analysis of a single group, which led to difficulties in comparing results in the study; the inability to assess patients soon after medical consultation, which would have enabled an analysis with less influence of drug treatment or non–drug-guided treatment by the physician; the heterogeneity in distribution of time of the disease between participants; and the inability to control for conservatory treatments previously performed by other health services.

To our knowledge, no other studies have used the WHOQOL-OLD instrument to assess QL of elderly women with knee OA.

Further studies are required, particularly those using specific instruments to evaluate an elderly population in order to understand the human aging process and take actions to benefit elderly people.

## CONCLUSION

We conclude that functional commitment due to knee OA could negatively affect the QL of elderly women. However, on the basis of the results of this study, physical domain should not be emphasized over other aspects of a person's life. Good QL is possible in elderly people, with or without disease.
